# Tracheobronchial calcification: an incidental finding in a patient on long-term Warfarin treatment

**DOI:** 10.11604/pamj.2024.48.127.41936

**Published:** 2024-07-24

**Authors:** Aishwarya Kishor Kedar, Vivek Dipakrao Alone

**Affiliations:** 1 Department of Respiratory Medicine, Datta Meghe Institute of Higher Education and Research, Wardha, Maharashtra, India

**Keywords:** Warfarin, tracheobronchial tree, calcification

## Images in medicine

A 90-year-old woman with nonvalvular chronic atrial fibrillation on Tab Warfarin since past 20 years came with complaints of right lower limb pain following a road traffic accident. Her vitals were within normal range. On right femoral anteroposterior radiograph, she was diagnosed to have right distal femur fracture. Patient was planned for open reduction and internal fixation for the same. Preoperative investigations were done which included chest radiograph, electrocardiogram and laboratory investigations. Chest radiograph revealed diffuse tracheobronchial tree calcification as an incidental finding. Patient had no respiratory complaints. Electrocardiogram showed atrial fibrillation. Laboratory testing revealed normal serum calcium levels of 8.9 mg/dl, serum phosphorous levels of 3 mg/dl, parathyroid hormone levels of 15 pg/ml and vitamin D levels of 50 ng/ml. Bone mineral density test revealed a t- score of -3 and a diagnosis of osteoporosis was made. Patient was operated for open reduction and internal fixation and given Tab Denosumab for osteoporosis. Patient was discharged in a vitally stable state with no change of medication of Warfarin. The association between chronic Warfarin therapy and progressive tracheobronchial calcification is well established. The mechanism by which Warfarin causes tracheal calcification is unknown, but it may inhibit the normal formation of vitamin K-dependent proteins that prevent cartilage and connective tissue from calcifying. This condition is distinct from tracheobronchial osteochondroplastica, a tracheobronchial calcification disease in the elderly in which the airways are unevenly affected and multiple calcified nodules form within the anterolateral wall of the trachea. It is important to exclude serum calcium and phosphorus imbalances, such as those seen in hyperparathyroidism. If airway calcification is localized or associated with tracheobronchial narrowing or wall thickness, relapsing polychondritis and amyloidosis should be considered. It is not a contraindication to continuing warfarin treatment. We report a case of tracheobronchial calcification identified as an incidental finding in a patient on long-term treatment with Warfarin for chronic atrial fibrillation.

**Figure 1:**
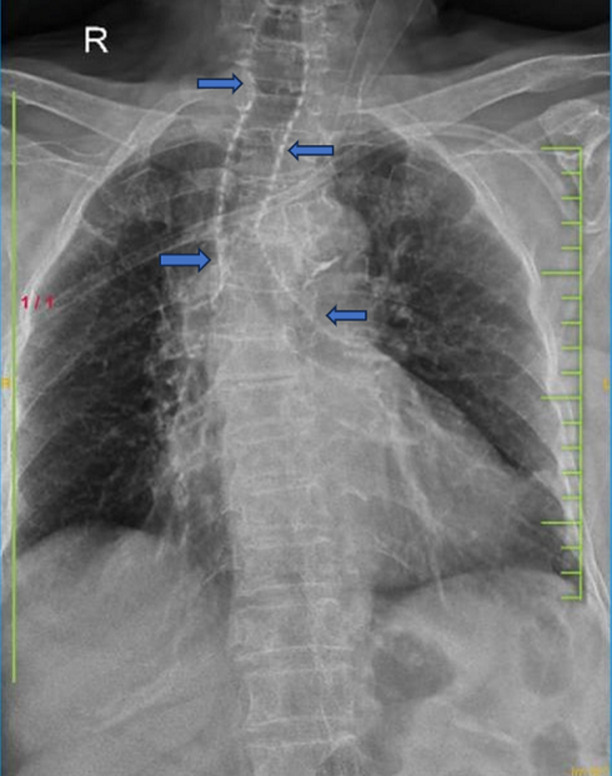
tracheobronchial calcification

